# Editorial: From black-box to clarity in lesion diagnostics: clinical causal cognition led interpretable diagnostic AI systems

**DOI:** 10.3389/fmed.2026.1962644

**Published:** 2026-09-02

**Authors:** Dong Zhang, Yucheng Guo, Xiaoxi Liu, Luping Lin

**Affiliations:** 1 Department of Ultrasound, the Second Affiliated Hospital of Xi'an Jiaotong University, and State Key Laboratory of Human-Machine Hybrid Augmented Intelligence, Institute of Artificial Intelligence and Robotics, Xi'an Jiaotong University, Xi'an, China; 2 Key Laboratory of Shaanxi Province for Craniofacial Precision Medicine Research, College of Stomatology, Xi'an Jiaotong University, Xi'an, China; 3 Laboratory of Statistical Genetics and Translational Medicine, Center for Life Medical Sciences, RIKEN Yokohama Campus, Yokohama, Japan; 4 School of Foreign Studies, Xi'an Jiaotong University, Xi'an, China

**Keywords:** causal cognition, hybrid augmented intelligence, interpretable diagnostics, lesion recognition, medical image analysis

Medical image analysis has made remarkable progress in lesion detection and characterization, yet deep learning models generally face the dual dilemma of “black-box decision-making” and “reliance on statistical correlations" (1–3). Conventional methods fit associations between imaging features and pathological outcomes using large-scale data, but struggle to disentangle the causal mechanisms underlying confounding factors, resulting in high misdiagnosis rates in complex clinical scenarios. More critically, existing models often overlook the alignment between model reasoning and clinical cognition, failing to construct interpretable pathways consistent with clinical thinking. What clinicians require goes far beyond feature heatmaps; they demand complete causal-sequential evidence. Against this backdrop, a novel diagnostic paradigm driven by clinical causal cognition has emerged as a crucial breakthrough direction.

This Research Topic was launched to address the inherent challenges of “black-box decision-making” and “overreliance on statistical correlations” in current medical image analysis models. It aims to explore hybrid intelligent systems that integrate clinical cognitive knowledge and proactive human–computer interaction, transforming imaging features into traceable causal chains to align AI decision logic with clinical cognition. The overarching goal is to establish a diagnostic paradigm where AI reasoning resonates with physicians’ clinical thinking, delivering more accurate and trustworthy support for clinical diagnosis. The topic solicits research contributions across six themes: interpretable AI in reducing the misdiagnosis rate of medical imaging; strategies for improving AI decision logic based on clinical cognition; integrating multimodal data (imaging, clinical, and multi-omics) for interpretable and context-aware AI diagnostics; pathophysiological knowledge-driven approaches for interpretable lesion recognition; human-computer collaboration frameworks for lesion identification and characterization; and graph network-based methods for interpretable diagnostic insights.

This topic includes 10 research papers covering multiple clinical scenarios including respiratory, renal, neurological, cardiovascular, gastrointestinal and endocrine diseases, addressing the core propositions of the Research Topic from diverse perspectives.

In the field of pulmonary disorders, Chen and Wu retrospectively analyzed chest CT findings in 50 patients with COVID-19, confirming that CT exhibits high sensitivity when nucleic acid testing is scarce. Specific CT phenotypes and severity scores can effectively predict disease progression and guide clinical management. Wu et al. proposed PulmoX-Net, a model combining depthwise separable convolutions with a channel attention mechanism, which achieved 89.23% accuracy in a 9-class chest radiograph classification task and provided visual interpretability via Grad-CAM heatmaps. Directly echoing the core theme of this collection, Li et al. constructed a concept bottleneck layer and a lesion-context graph network, realizing pathophysiologically consistent causal reasoning pathways for predicting complications of microwave ablation for lung cancer. They verified causal consistency through monotonicity tests and counterfactual analysis, significantly improving the usability of human–computer collaboration.

In abdominal and cardiovascular diseases, Zhang et al. combined conventional ultrasound with point shear wave elastography (pSWE) for chronic kidney disease assessment, finding that renal tissue elasticity increases with disease stage and is highly correlated with the degree of interstitial fibrosis, providing noninvasive quantitative biomarkers for different pathological subtypes. Cheng et al. developed a nomogram for predicting the risk of pathological upgrading of colorectal polyps based on five endoscopic morphological features, with an AUC of 0.922. They enhanced model interpretability using the SHAP method to support individualized treatment decisions. Gao et al. compared the performance of deep learning and traditional machine learning in stroke risk stratification of carotid plaques, confirming that the ResNet-50 model achieved an AUC of 0.982, significantly outperforming conventional algorithms. Mai et al. integrated transthoracic echocardiography and clinical data to construct a left atrial appendage thrombosis prediction model, with the logistic regression algorithm reaching an AUC of 80.9%, providing a noninvasive screening tool for high-risk patients with atrial fibrillation.

In multimodal and neuroscience fields, Ruan et al. established an auxiliary diagnostic model for Hashimoto's thyroiditis based on tongue manifestation features, with the SVM algorithm achieving an AUC of 0.894, offering a low-cost noninvasive screening approach for primary care settings. Han et al. proposed a unified multi-view hypergraph learning framework that integrates knowledge-driven and data-driven strategies. It captures high-order associations among brain regions in the diagnosis of neurodevelopmental disorders, providing new insights into understanding disease pathogenesis.

Overall, the studies included in this topic jointly advance diagnostic AI from “black-box correlation” to “transparent causality” across multiple dimensions: imaging feature optimization, multimodal data fusion, causal pathway construction, and human–computer collaboration mechanisms. These findings not only validate the clinical value of interpretable AI across various specialty scenarios, but also lay the foundation for building a unified clinical causal cognition diagnostic framework. Looking ahead, with the deep integration of pathophysiological knowledge and continuous improvement of causal reasoning methods, AI systems will further achieve deep alignment between decision logic and clinical thinking, ultimately moving toward a more trustworthy and efficient era of intelligent diagnosis.
